# Validation of the Italian version of the Laval questionnaire: health-related quality of life in subjects with obesity

**DOI:** 10.1186/s12955-017-0671-3

**Published:** 2017-05-15

**Authors:** Lorenzo Maria Donini, Aldo Rosano, Luca Di Lazzaro, Eleonora Poggiogalle, Carla Lubrano, Silvia Migliaccio, Mariagrazia Carbonelli, Alessandro Pinto, Andrea Lenzi

**Affiliations:** 1 High Specialization Centre for the Care of Obesity (“CASCO”), Department of Experimental Medicine, Medical Pathophysiology, Food Science and Endocrinology Section, “Sapienza” University of Rome, Rome, Italy; 2 Istituto Superiore di Sanità, Rome, Italy; 3 ”Foro Italico” University of Rome, Rome, Italy; 4 “S. Camillo-Forlanini” Hospital, Rome, Italy; 5 “Sapienza” University of Rome, Department of Experimental Medicine- Medical Pathophysiology, Food Science and Endocrinology Section, Food Science and Human Research Unit, p.le Aldo Moro n.5, 00185 Rome, Italy

**Keywords:** Quality of life, Obesity

## Abstract

**Background:**

Obesity is associated to increased risk of metabolic comorbidity as well as increased mortality. Notably, obesity is also associated to the impairment of the psychological status and of quality of life.

Only three questionnaires are available in the Italian language evaluating the health-related quality of life in subjects with obesity.

The aim of the present study was to test the validity and reliability of the Italian version of the Laval Questionnaire.

**Methods:**

The original French version was translated into Italian and back-translated by a French native speaker. 273 subjects with obesity (Body Mass Index ≥ 30 kg/m^2^) were enrolled; the Italian version of the Laval Questionnaire and the O.R.Well-97 questionnaire were administered in order to assess health- related quality of life. The Laval questionnaire consists of 44 items distributed in 6 domains (symptoms, activity/mobility, personal hygiene/clothing, emotions, social interaction, sexual life). Disability and overall psychopathology levels were assessed through the TSD-OC test (SIO test for obesity correlated disabilities) and the SCL-90 (Symptom Checklist-90) questionnaire, respectively. To verify the validity of the Italian version, the analysis of internal consistency, test-retest reliability, and construct validity were performed.

**Results:**

The observed proportion of agreement concordance of results was 50.2% with Cohen’s K = 0.336 (CI 95%: 0.267–0.404), indicating a fair agreement between the two tests.

Test-retest correlation was statistically significant (ρ = 0.82; *p* < 0.01); validity (standardized Chronbach’s alpha) was considered reliable (α > 0.70). The analysis of construct validity showed a statistically significant association in terms of both total score (ρ = −0.66) and scores at each single domain (*p* < 0.01). A high correlation (*p* < 0.01) was observed between Laval questionnaire total and single domain scores and other related measures (Body Mass Index, TSD-OC scores, SCL-90 global severity index), revealing a high construct validity of the test.

**Conclusions:**

The Italian version of the Laval Questionnaire is a valid and reliable measure to assess the health-related quality of life in subjects with obesity.

## Background

Defined as excess fat mass, and mirrored by the increase of the body mass index, obesity is a multifactorial, chronic disease [1]. A wealth of studies showed that obesity is associated to increased morbidity (especially cardiovascular, respiratory, metabolic, osteoarticular, and neoplastic diseases) as well as to higher all- cause mortality [2].

In Europe obesity accounts for 2– 8% of health care costs and for 10– 13% of mortality. According to data from the WHO, in the European region the prevalence of overweight and obesity is 25– 79% and 5– 30% respectively in the adult population [3, 4].

Furthermore, the presence of obesity deeply affects both physical and psychosocial functioning, leading to the onset of disability, especially difficulty in the activities of daily living, indoor mobility, housework and domestic activities, outdoor activities and occupational activities [5, 6]. Hence, the quality of life is significantly reduced in subjects with obesity [5–7].

Despite a number of instruments have been developed in order to evaluate the health- related quality of life [8], just few tools exist currently for the selective assessment of the health- related quality of life in the obese population [9–16], and just one of them has been developed and validated in Italy, namely the Obesity Related Wellbeing (O.R.Well-97) questionnaire [17]. Save the Impact of Weight on Quality of Life (IWQOL) [14] and the IWQOL-Lite [15] questionnaires, the remaining above- mentioned tools have not been translated into Italian.

In the most used questionnaires for the assessment of the health- related quality of life, dimensions usually explore physical health (symptoms, pain, function and functional limitations), mental health (behavior alterations and emotional status), social activities, working performance, and self- perception of the health status [18–22].

In addition to aspects related to physical health and to the psychosocial domain, in subjects with obesity other features are also taken into account: the different degrees of disability due to excess body weight (functionality and autonomy in the activities of daily living, in indoor and outdoor activities, occupational activities and social life), the level of mobility, personal care and hygiene, and sexual life [18–22].

Beside the clinical indicators, in a health-related quality of life questionnaire, as well as in other types of measures, other characteristics are required: especially in terms of validity, reliability, internal consistency, ability to detect statistically significant differences between groups and over time, and interpretability of the results. These requirements are pivotal and need to be maintained when questionnaires are translated and validated in different linguistic and cultural settings [23].

Recently a new tool investigating quality of life in individuals with obesity has been validated and published: the Laval questionnaire [16], developed in the bariatric surgery unit at the Laval Hospital in Quebec City, Canada.

The aim of the present study was to translate and to validate the Laval questionnaire for the Italian obese population.

## Methods

### Translation

Originally developed in French, the Laval questionnaire was translated from French to Italian using a standard forward- backward translation procedure. The French version of the Laval questionnaire was first translated into Italian, and then back- translated into French (by a native French speaker without reference to the French original version).

### Population study

Between June 2012 and August 2016, 273 subjects with obesity (199 women and 74 men) were recruited among outpatients referring to the High Specialization Centre for the Care of Obesity (named “CASCO”) at the Department of Experimental Medicine- Pathophysiology, Food Science and Endocrinology Section, “Umberto I” University Hospital - Sapienza University of Rome, Italy, and to the “S. Camillo” Hospital, Rome, Italy. Inclusion criteria were the ability to follow instructions to fill out the questionnaires, BMI ≥ 30 Kg/m^2^ and age ≥ 18 and < 75 years. Similar to the validation study of the Laval questionnaire [16], there was no exclusion criteria concerning the presence of comorbidities (such as obstructive sleep apnea, diabetes or osteoarthritis) except for severe psychiatric disorders.

### Demographics and clinical status

Medical history, including obesity-related complications (insulin resistance, type 2 diabetes, hypertension, dyslipidemia, thyroid dysfunction, cardiovascular, osteoarticular, respiratory, gastrointestinal, and psychiatric diseases), as well as demographic, social and cultural information (age, gender, occupational position and education level) were obtained.

### Anthropometric measurements

All subjects underwent physical examination. Anthropometric measurements were gathered following standardized procedures [24]. Body weight was measured using a SECA scale (Hamburg, Germany) to the nearest 0.1 kg; height was measured using a SECA stadiometer (Hamburg, Germany) to the nearest 0.5 cm. Body mass index [BMI = body weight (kg)/height squared (m^2^)] was calculated.

### Questionnaires

Two health-related quality of life questionnaires, the Laval questionnaire [16], and the O.R.WELL-97 questionnaire [17] were self- administered, at the time of the first access to our center (T-1), and 1 week after the first access (T-2):

- The Laval questionnaire consists of 44 items distributed in 6 domains (symptoms, activity/mobility, personal hygiene/clothing, emotions, social interaction, and sexual life). Each domain is scored on a 7- point Likert scale, with higher scores corresponding to a better quality of life [16].
- The O.R.Well- 97 questionnaire is composed of 18 items; for each item the patient is asked to score on a 4- point Likert scale the occurrence and/or severity of the symptom (occurrence) and the subjective relevance of the symptom- related impairment in one’s own life (relevance) [17].

In addition two more questionnaires were administered:

- TSD- OC (SIO test for obesity- related disabilities) is a reliable and valid instrument for measuring self-reported disability in subjects with obesity. The questionnaire consists of multiple questions in 7 dimensions to measure pain, stiffness, functionality and autonomy in daily activities, housework, outdoor activities, occupational activities, and social life. Each question is scored by the subject from 0 (lack of disability) to 10 (maximum disability) [25].
- SCL- 90 (the Symptom Checklist-90) is a brief self- report psychometric questionnaire designed to evaluate several psychological problems and symptoms of psychopathology. It consists of 90 items, summarized in a Global Severity Index (GSI) [26].

### Statistics

Continuous variables were examined for skewness and kurtosis and distributional assumptions were verified. The Spearman’s ρ statistics was used to evaluate the rank-order association between two ordinal variables.

To validate the Italian version of the Laval questionnaire:

- Cohen’s kappa coefficient was used to measure agreement between two raters for qualitative (categorical) items. For this purpose the O.R.Well-97 and Laval questionnaire scores were split in quartiles. Cohen suggested the Kappa result to be interpreted as follows: values ≤ 0 as indicating no agreement;0.01– 0.20 as none to slight; 0.21–0.40 as fair; 0.41– 0.60 as moderate; 0.61–0.80 as substantial; and 0.81–1.00 as almost perfect agreement [27];
- Cronbach’s Alpha standardized model based on average correlations between elements was used to analyze the reliability of the test based on the degree of correlation between elements of the questionnaire. Dimensions were considered reliable if α > 0.70 [28];
- Test- retest reliability of the Laval questionnaire was determined by correlating the results obtained at T1- and at T-2 assuming clinical ad functional stability;
- Construct validity was evaluated through the Spearman’s ρ statistics between the Italian version of Laval Questionnaire total score and the O.R. Well- 97 questionnaire total score. The correlation between scores for every single domain in the two questionnaires was also calculated;
- Discriminative property of the Italian version of the Laval Questionnaire was evaluated correlating the total score and the scores of single domains of the questionnaire with other related measures (BMI, TSD-OC score, SCL-90 global severity index).

Differences were considered to be statistically significant for *p* < 0.05. Statistical analysis was performed using SPSS statistical software (IBM Corp. Released 2013. IBM SPSS Statistics for Windows. Armonk, NY: IBM Corp).

## Results

Two hundred seventy-three subjects (74 men and 199 women) were enrolled. Demographic and clinical characteristics are summarized in Table 1.

**Table 1:** Participants’ clinical and demographic characteristics

		Men (*n* = 74)	Women (*n* = 199)
Age (years)		46.2 ± 14.2	46 ± 13.5
BMI (kg/m^2^)		40.4 ± 8.3	34.8 ± 6.2
Obesity-related diseases (%)	Type 2 Diabetes	14.9	12.6
	Thyroid diseases	6.8	35.2
	Gastrointestinal diseases	23	19.6
	Cardiovascular diseases	16.2	5.0
	Hypertension	45.9	36.2
	Osteaorticular diseases	25.7	22.1
	Respiratory diseases	21.6	16.6
	Psychiatric diseases	14.9	12.1
	Dyslipidemia	16.2	12.6
	Insulin resistance	10.8	15.1
Level of education (%)	Primary school	2.7	5
	Middle school	20.3	25.3
	High school	60.8	55.1
	University degree	16.2	14.6
Occupation (%)	Housewife	1.4	32,7
	Employee	21.9	29.6
	Freelance worker	17.6	10.1
	Factory worker	14.9	8.5
	Retiree	12.2	9.5
	Unemployed	6.8	5.5
	Student	5.4	4

*BMI* Body mass index

The observed proportion of agreement concordance of results was 50.2% with Cohen's Kappa = 0.336 (CI 95%: 0.267–0.404), indicating a fair agreement between the two tests.

Test- retest correlation for the Laval questionnaire is presented in Table 2; the correlation between the test and retest total scores obtained at the first and second administration respectively was statistically significant (ρ = 0.82; *p* < 0.01), as well as results were significant (*p* < 0.01) for each single domain [Symptoms (ρ = 0.88), Activity/mobility (ρ = 0.94), Personal hygiene/clothing (ρ = 0.91), Emotions (ρ = 0.9), Social interactions (ρ = 0.87), Sexual life (ρ = 0.85)].

**Table 2:** Test-retest reliability

Correlation between test at time 1 vs. retest at time 2	ρ	*p*
Total score	0.82	*
Activity/mobility	0.73	*
Symptoms	0.76	*
Personal hygiene- clothing	0.72	*
Emotions	0.69	*
Social interactions	0.69	*
Sexual life	0.59	*

*Time 1* first access to our center, *Time 2* 1 week after the first access

*Each correlation (Spearman’s ρ) between test and retest scores was statistically significant (*p* < 0.01)

Construct validity of the Italian version of the Laval questionnaire was obtained by correlating the total score with the scores of each section (standardised Chronbach’s alpha). All dimensions were considered reliable (α > 0.70): 0.87 for symptoms, 0.9 for activity/mobility, 0.88 for personal hygiene/clothing, 0,85 for emotions, 0.84 for social interactions and 0.79 for sexual life.

Construct validity: as described in Table 3, when compared to the O.R.Well-97 test, the Laval questionnaire showed a statistically significant association in terms of both total score (ρ = − 0.66) and scores at each single domain. The scores obtained at the four sections of the O.R.Well-97 questionnaire (occurrence, relevance, psychosocial status-adjustment, physical discomfort) were all highly significantly correlated (*p* <0.01) with the scores obtained at the six sections of the Laval questionnaire (activity- mobility, symptoms, personal hygiene- clothing, emotions, social interactions).

**Table 3:** Correlation at time 1 between Laval e O.R.Well-97 questionnaires (convergent validity)

	O.R.Well				
	Total score	Occurrence	Relevance	Psychosocial status-psychosocial adjustment	Physical discomfort
Laval					
Total score	−0.66	−0.56	−0.66	−0.38	−0.41
Activity-mobility	−0.53	−0.41	−0.56	−0.28	−0.39
Symptoms	−0.46	−0.39	−0.45	−0.29	−0.39
Personal hygiene-clothing	−0.67	−0.52	−0.58	−0.37	−0.33
Emotions	−0.6	−0.53	−0.39	−0.27	−0.24
Social interactions	−0.6	−0.39	−0.54	−0.18	−0.19
Sexual life	−0.53	−0.4	−0.58	−0.25	−0.19

N.B.: Each correlation (Spearman’s ρ) between Laval and O.R.Well-97 scores was statistically significant (*p* < 0.01)

The observed cross- sectional correlations analyzing the discriminative validity of the Laval questionnaire are shown in Table 4. A highly significant correlation was observed between Laval questionnaire (total and single domain scores) and other related measures (BMI, TSD-OC scores, SCL-90 global severity index) ranging from ρ = − 0.2 to 0.4 for BMI and from − 0.5 to − 0.7 for SCL-90, thus revealing a high construct validity of the test.

**Table 4:** Discriminative properties (concurrent validity) of the Italian version of the Laval questionnaire

Laval	BMI	TSD-OC								SCL-90 (GSI)
		Tot	Pain	Stiffness	FADA	House-work	Outdoor	Occup	Social	
Total score	−0.41	−0.69	−0.6	−0.57	−0.63	−0.63	−0.59	−0.52	−0.58	−0.7
Activity-mobility	−0.4	−0.76	−0.67	−0.6	−0.73	−0.68	−0.64	0.53	−0.6	−0.58
Symptoms	−0.34	−0.63	−0.61	−0.5	−0.59	−0.57	−0.51	−0.44	−0.47	−0.6
Personal hygiene-clothing	−0.37	−0.64	−0.54	−0.5	−0.67	−0.57	−0.51	−0.46	−0.57	−0.54
Emotions	−0.29	−0.51	−0.42	−0.42	−0.43	−0.46	−0.45	−0.41	−0.49	−0.71
Social interactions	−0.37	−0.6	−0.48	−0.47	−0.57	−0.53	−0.52	−0.43	−0.6	0.61
Sexual life	−0.24	−0.5	−0.39	−0.4	−0.43	−0.44	−0.41	−0.37	−0.46	−0.52

*BMI* body mass index, *SCL-90* Symptom Checklist-90, *GSI* global severity index

TSD-OC (SIO test for obesity related disabilities) categories: pain; stiffness; FADA: function and autonomy in daily activities; housework; outdoor activities; occupational activities; social life

N.B.: Each correlation (Spearman’s ρ) between Laval and other related measures was statistically significant (*p* < 0.01)

## Discussion

The Laval questionnaire is a 44- item measure of health- related quality of life validated in subjects with obesity, developed primarily for use in bariatric surgery clinical practice [16]. This study supports the validity of the Italian version of the Laval questionnaire in subjects with obesity seeking for either conservative therapy for obesity or bariatric surgery.

Indeed, the analysis of internal consistency and the test- retest reliability confirm the intrinsic validity of the test in the Italian version. Moreover, the construct validity through the correlation between the Italian version of Laval Questionnaire and O.R.Well-97 questionnaire (both total score and every single domain) confirms the ability of the test to assess the health- related quality of life in subjects with obesity.

The construct capacity of the test was confirmed through the analysis of the correlation between Laval questionnaire total and single domain scores and other related measures (BMI, TSD-OC scores, SCL-90 global severity index).

The Laval questionnaire score was significantly correlated with the TSD-OC test score, that is a specific tool for the evaluation of disability in subjects with obesity. In fact, a peculiar characteristic of the Laval questionnaire is represented by the fact that it considers, when compared to other similar tests, not only social interactions, sexual life, emotions and symptoms (as the only other tool currently available in Italian: the O.R.Well-97 questionnaire), but emphasis is also placed on the limitations to the activities of daily living (ADL). Independence, psychological wellbeing and overall quality of life at all ages are affected in obesity due to clinical implications, such as comorbidities and somatic fragility [29, 30]. The 2000–2001 Health Survey for England [31] showed that obesity was significantly correlated with many disabling conditions, such as osteoarthritis, diabetes mellitus and chronic obstructive pulmonary disease. Moreover, these diseases, often associated with obesity, are considered among the 10 most troublesome diseases for high- income countries in the WHO’s Global Burden of Disease study [32]. Obesity is characterized by an increased risk of disability (limited mobility and activities of daily living) and by a reduced number of disability-free years (5.7 years for men and 5.02 years for women) [33]. Impairment in ADLs (i.e. personal hygiene and dressing), indoor mobility, household chores (i.e. rising from a couch, getting onto a high stool and taking objects from a cupboard or picking them up from the floor), outdoor activities (i.e. lifting and carrying bags, walking for 100 m and standing in a queue) and occupational activities are frequent in subjects with obesity [34–43].

Impairment of the health- related quality of life in subjects with obesity was observed also in relation to increasing BMI class and construct psychopathology in different studies [44–46]. In the original validation study of the Laval questionnaire [16], the Authors found a significant correlation between the score at the Beck Depression Inventory and the “Emotions” domain of the Laval. In a study conducted by Mannucci et al. [46] at the multivariate analysis, psychopathology (presence of previously-diagnosed mental disorders and/or elevated scores on SCL- 90) was associated with lower quality of life scores in both psychosocial and somatic domains. Similarly, in our study cohort all domains and theglobal score of the Laval questionnaire were significantly correlated with the global severity index of SCL- 90.

In the original validation study of the Laval questionnaire the Authors used the Impact of Weight on Quality of Life-lite (IWQOL-Lite) test [14]while in our study we used the O.R.Well- 97 questionnaire [17] as anotherquestionnaire measuring constructs related to quality of life, given that this second test was validated in an Italian population of outpatients seeking treatment for obesity. We chose an already validated measure in Italian in order to minimize the effects related to the cultural and behavioral diversity that might be present between the populations in which the different tests were validated.

The question may arise whether to translate a new test into Italian since there are already two questionnaires (O.R.Well-97 and the IWQOL-Lite) available in Italian. Compared to these two tests, The Laval Questionnaire has a greater ability to assess the multiple factors contributing to the quality of life in individuals with obesity: it explores six different dimensions (activity- mobility, symptoms, personal hygiene- clothing, emotions, social interactions and sexual life) while O.R.Well-97 takes into account “only” the occurrence, the relevance, the psychosocial-status, and the physical discomfort related to obesity. On the other hand, IWQOL-Lite limits its analysis to physical functionality, self- esteem, sexual life, public distress, and work, considering, to a lesser extent, emotions, personal- hygiene and clothing. Moreover, the fact that these two tests have a lower number of items than the Laval Questionnaire does not seem to be of particular relevance, given that the three tests are all self-administered and they do not affect significantly the workload and time of health-care professionals.

The main limitation to our study is represented by the study population who was a convenience sample of subjects with obesity; however, they were recruited, as for the validation study of the Laval questionnaire [16], without any exclusion concerning BMI and the presence of comorbidities (such as obstructive sleep apnea, diabetes or osteoarthritis).

## Conclusions

The Italian version of Laval Questionnaire has been demonstrated to maintain the reliability and validity of the original version, allowing a thorough evaluation of the impact of obesity on the health status and its consequences in terms of quality of life.

The responsiveness of the Italian version of the Laval questionnaire needs to be further verified in measuring changes in health-related quality of life associated to obesity treatment.

## Abbreviations

ADL: Acitivities of Daily Living
BMI: Body Mass Index
CASCO: High Specialization Centre for the Care of Obesity
GSI: Global Severity Index
IWQOL: Impact of weight on quality of life
O.R.Well-97: Obesity Related Wellbeing
SCL-90: Symptom Checklist-90
TSD-OC test: SIO Test for Obesity Correlated Disabilities
